# Evolution of Compaction Characteristics and Void Features in Stone Mastic Asphalt Mixtures Based on Computed Tomography Images

**DOI:** 10.3390/ma18071513

**Published:** 2025-03-27

**Authors:** Xia Wu, Zhaoyi He, Maorong Li, Tiang Tang, Dingbang Wei

**Affiliations:** 1 School of Civil Engineering, Chongqing Jiaotong University, Chongqing 400074, China; xswuxia1@jxyy.edu.cn (X.W.);; 2 School of Architecture and Engineering, Jiangxi College of Applied Technology, Ganzhou 341000, China; 3 Gansu Transport Planning, Survey and Design Institute Co., Ltd., Lanzhou 730030, China; kzxian029@126.com; 4 Gansu Province Highway Traffic Construction Group Co., Ltd., Lanzhou 730030, China

**Keywords:** road engineering, stone mastic asphalt, compaction properties, CT images, void characteristics, correlation

## Abstract

To investigate the dynamic evolution of macro- and fine-scale characteristics during the compaction process of Stone Mastic Asphalt (SMA-13), the following methodology was employed. First, the compaction characteristics were analyzed based on the Marshall compaction test, and an exponential regression model for compaction was established. A compaction coefficient was proposed to evaluate the ease of compaction of SMA-13. Second, Marshall specimens subjected to different number of blows (5, 15, 25, 50, 75, 100, and 125 times per face) were scanned using a Computed Tomography (CT) scanner, and image processing techniques were applied to precisely extract void characteristics (void number, void area, and equivalent void diameter) under various compaction states. Third, the evolution of void characteristics in SMA-13 during the compaction process was analyzed, and correlations with macroscopic compaction properties were established. The experimental results demonstrated that SMA-13 achieved optimal compaction at an asphalt content of 5.9% and an initial compaction temperature of 170 °C. The established compaction numerical regression model can effectively characterize the compaction characteristics of SMA-13. A higher compaction coefficient indicated easier compaction and better compaction performance. During compaction of SMA-13, the void number and void area exhibited exponential decay, and voids with equivalent diameters of 1–7 mm gradually decreased, showing a non-proportional linear decay in their distribution. In contrast, voids with equivalent diameters of 0–1 mm increased during compaction, and they are the dominant component of the void structure in SMA-13. During compaction, the void ratio of SMA-13 gradually decreased along the direction of height, and the distribution of void ratio was “great at both ends and small in the middle”. The void ratio at 5–55 mm decreased from about 10% to about 0%, and the void ratio distribution was relatively uniform. The void ratio at the bottom 0–5 mm and top > 50 mm was large and unevenly distributed, mostly between 10% and 40%.

## 1. Introduction

Asphalt mixtures are composite, dense, and heterogeneous materials composed of asphalt, aggregates, and voids [1,2]. Stone Mastic Asphalt (SMA-13), characterized by its excellent skid resistance and durability, is widely used in the surface layer of asphalt pavements. It belongs to the category of gap-graded, skeleton-dense structures [3,4]. To ensure the quality and longevity of asphalt pavements, the compaction process is one of the critical control steps [5,6]. Currently, the compaction degree is a key indicator for assessing pavement compaction quality, and it is closely related to void ratio, void structure composition, and void morphology [7,8]. Researchers have conducted extensive studies on the void structure of formed asphalt mixtures using CT scanning techniques. These studies have focused on parameters such as CT-derived void ratio, void number, void area, equivalent void diameter, and microscopic void morphology (e.g., cross-sectional roundness, cross-sectional fullness, void angle, and principal axis orientation) [9,10,11,12,13].

Studies have shown that the Marshall variable compaction test can effectively reflect the compaction process of asphalt pavements [8,11]. The evolution of key parameters such as void ratio, compaction degree, and compaction curves provides valuable insights into the mechanisms of the compaction process [14,15,16,17,18]. CT scanning combined with MATLAB v. 2024 image processing techniques can accurately characterize the fine-scale structure of asphalt mixtures, enabling the precise investigation into fine-scale void volume distribution [19,20,21,22,23,24,25]. Significant changes in void ratio, void number, and void equivalent diameter have been observed in asphalt mixtures under different compaction work [11]. Compaction primarily affects the volume-related indicators of voids (e.g., void ratio, void number, and void equivalent diameter) rather than their morphological characteristics [8]. Additionally, asphalt content and compaction temperature significantly influence the compaction quality of asphalt mixtures during compaction [26,27].

While previous research has primarily focused on the fine-scale void characteristics of formed asphalt mixtures, there is limited investigation into the dynamic changes in void characteristics within the asphalt mixture during compaction. Moreover, few studies have explored the correlation between void characteristic evolution and macroscopic compaction properties.

Therefore, this study investigates the compaction characteristics of SMA-13 (e.g., compaction degree, compaction curves, compaction regression models, and compaction coefficient) based on compaction tests. Additionally, the dynamic evolution of void fine-scale indicators is analyzed based on CT scanning and image processing techniques, and correlations with compaction characteristics are established. This approach helps to establish a logical relationship between the macroscopic compaction features and fine-scale void indicators of asphalt mixtures, providing new insights and methodologies for understanding the compaction mechanisms of asphalt mixtures.

## 2. Materials and Methods

### 2.1. Materials

In this study, SMA-13 was selected as the research subject. The mixture was prepared using 90# SBS modified asphalt, the technical properties of which are listed in Table 1. The aggregates were sourced from basalt quarries in Sichuan Yingjing Kaiquan Industrial Co., Ltd., Sichuan, China, with the technical properties of coarse aggregates, fine aggregates, and mineral powder provided in Table 2. The aggregate gradation is detailed in Table 3. Additionally, lignin fiber was incorporated at a content of 0.4%, with a relative density of 1.2.

The incorporation of lignin fiber (0.4% by weight) was aimed at enhancing the mixture’s stability and durability, with its relative density measured at 1.2. This combination of materials was designed to ensure optimal performance in terms of compaction, void structure, and mechanical properties for SMA-13.

### 2.2. Methods

To investigate the dynamic compaction characteristics and fine-scale void change features of SMA-13, the test scheme consisted of five sections: Marshall specimen preparation, CT scanning, digital image processing, compaction degree definition, and void feature calculation.

#### 2.2.1. Preparation of Marshall Specimens with a Variable Number of Blows

The Marshall compaction test is the standard indoor molding method for asphalt mixtures. The Marshall test is designed based on the equivalent compaction energy on site. Currently, SMA-13 indoor specimens are generally molded by the Marshall test. Engineering practice and research have shown that the dense structure formed in the Marshall test has a high similarity to the compacted structure on site [8,11]. Therefore, SMA-13 specimens with a variable number of blows were prepared to simulate the compaction process on site.

First, based on the SMA-13 target void ratio of 3.5%, the optimum aggregate gradation and optimum asphalt content were determined using the Marshall design method. The optimum aggregate gradation of SMA-13 is shown in Table 3. The optimum asphalt content of SMA-13 was 5.9% (±0.1%). Next, the optimum initial compaction temperature was determined to be 169 °C (±1 °C) by experimental tests with different initial compaction temperatures. Again, SMA-13 Marshall specimens were prepared based on an optimum initial compaction temperature of 170 °C and an optimum asphalt content of 5.9%. These specimens were molded under the same number of blows per face, such as 5, 15, 25, 50, 75, 100, and 125 per face. Thus, a total of six sets of specimens were prepared, with four parallel specimens in each set. Finally, these specimens were used to obtain compaction curves and analyze compaction properties. These specimens were scanned using a CT scanner to obtain the digital images, and the fine-scale void characteristics of each set of specimens were extracted based on the Digital Image Processing Technology.

In this way, it was possible to analyze the dynamic evolution of the void characteristics during the compaction process and to establish a correlation with the compaction characteristics.

To investigate the effects of asphalt content and initial compaction temperature on the compaction characteristics of SMA-13, specimens were prepared under identical aggregate gradation conditions with varying asphalt content (5.4%, 5.9%, and 6.4%) and initial compaction temperatures (150 °C, 170 °C, and 180 °C). These specimens were molded under the same number of blows per face, such as 5, 15, 25, 50, 75, 100, and 125 per face, at the same temperature or asphalt content. Each group of specimens had four parallel specimens, and the compaction degree was averaged with four specimens with an error of less than 5%.

#### 2.2.2. Computerized Tomography (CT) Scanning Test

CT Scanning Test is a non-destructive testing technique that enables the detection of the internal micro-structure of materials by leveraging differences in X-ray absorption across materials of varying densities. This method generates digital images by sampling intensity values at discrete points, allowing the acquisition of structural characteristics within the material.

**Figure 1:**
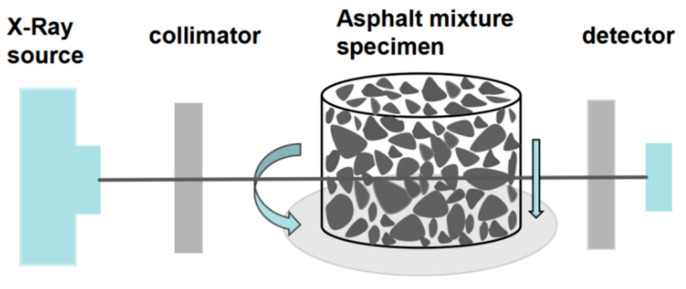
Schematic diagram of CT scanning principles.

In this study, a Phoenix v|tome|x S240 CT scanner (Baker Hughes, Houston, TX, USA) was utilized with the following parameters: Voltage: 190 kV, Current: 250 µA, and Resolution: 101 µm. The scanner captured raw slice images of the specimens (such as Figure 1).

In this study, CT scanning was used to analyze the laws of dynamic evolution of the fine-scale void structure during compaction of SMA-13. Prior to scanning, Marshall specimens with a diameter of 101.6 mm and a height of 63.5 mm were polished on both the top and bottom surfaces to minimize surface irregularities, resulting in a final height of approximately 60 mm (±1 mm).

Based on the method of specimen preparation in Section 2.2.1, the specimens molded with different numbers of blows (5, 15, 25, 50, 75, 100, 125 per face) under the optimal asphalt dosage and optimal initial temperature were subjected to CT scanning, and the CT images of all specimens were obtained. There were four parallel specimens in each group.

**Figure 2:**
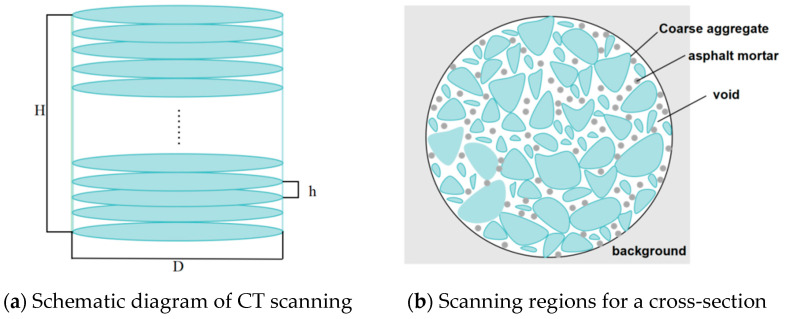
Schematic diagram of a CT scan.

The schematic diagram of the CT scanning is shown in Figure 2a, and the scanning regions for each cross-section are illustrated in Figure 2b.

In Figure 2a, H denotes the height of the scanned specimen, and D represents its diameter. With a scanning interval of 0.1 mm, approximately 600 images were acquired per specimen. Figure 2b illustrates the scanning regions for any cross-section. The asphalt mixture was composed of three primary components: coarse aggregates, asphalt mortar, and voids. During subsequent digital image processing, void features of these specimens were precisely identified and extracted.

#### 2.2.3. Digital Image Processing and Void Identification

**Figure 3:**
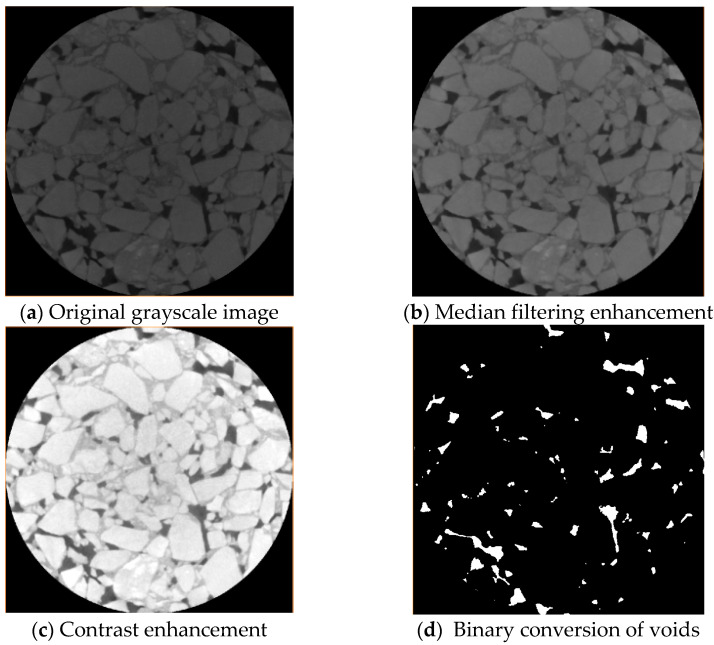
SMA-13 Gap Recognition Digital Image Processing Process.

In digital images of asphalt mixtures, aggregates appear nearly white, asphalt mortar appears gray, and voids are represented as black areas. Therefore, voids were treated as the target objects, while aggregates and asphalt mortar were considered as the background. The original image is shown in Figure 3a.

To ensure the accuracy of void identification in asphalt mixtures, the CT digital images of the asphalt mixture were processed as follows:

First, the original images were converted to 8-bit grayscale images by importing them into MATLAB v. 2024, as shown in Figure 3b.

Second, the images of the asphalt mixture were enhanced with a median filter, which can overcome the image blurring caused by linear filtering. The median filter is most effective in removing impulse interference and scanning noise for better image quality. Considering the small scanning noise, in order to preserve the clearer edge information, a 3 × 3 2D median filter was used for image processing, which was more effective, as shown in Figure 3c.

Third, the contrast between the voids and the aggregates and asphalt mortar was enhanced using adaptive histogram equalization, and the contrast-enhanced image is shown in Figure 3d.

Finally, after defining a fixed region of interest (ROI), the OTSU algorithm was employed for adaptive threshold segmentation. This method separates the target objects (voids) from the background (asphalt mortar and aggregates), as illustrated in Figure 3d. The OTSU algorithm dynamically determined the optimal threshold for void segmentation, improving accuracy in heterogeneous regions.

**Figure 4:**
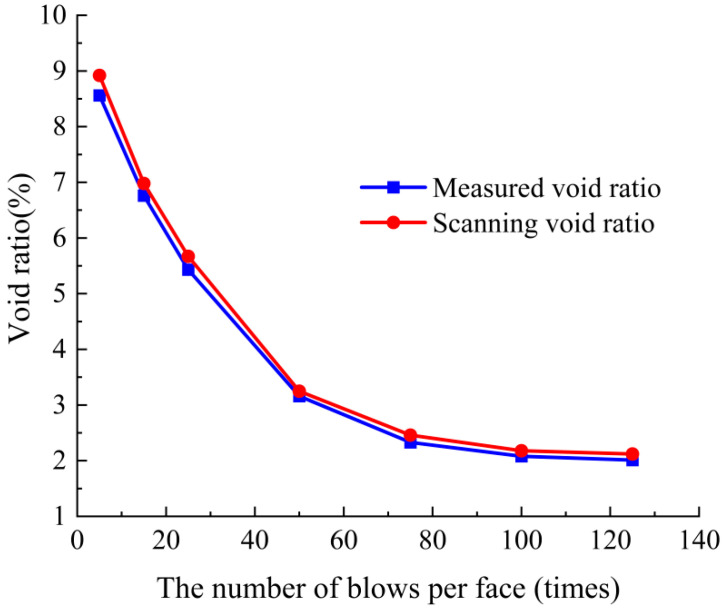
Comparison of the measured and scanning void ratio of SMA-13.

The scanning void ratio of the specimen was calculated based on Equation (6) in Section 2.2.5. The measured void ratio of the specimen was calculated according to Equations (2) and (3) in Section 2.2.4. Comparing the scanning void ratio of the specimen with the measured void ratio (shown as Figure 4), the average error was 4.37%. Therefore, the void characteristics obtained by the above image processing are valid.

This systematic image processing framework enabled robust quantification of void characteristics (e.g., number, area, equivalent diameter) of specimens, facilitating a comprehensive analysis of SMA-13’s void fine-scale structural evolution during compaction.

#### 2.2.4. Define the Compaction Degree and Void Ratio

Compaction degree (K) is an evaluation indicator that reflects the asphalt mixture’s compaction quality. In the indoor experiments, the compaction degree (K) of the asphalt mixture is the ratio of the bulk specific gravity to the maximum theoretical specific gravity for the specimen (dimensionless). The calculation formula is presented as follows:

(1) $$K=\frac{\gamma_{a}}{\gamma_{s}}\times 100\%$$

where $\gamma_{s}$ is the maximum theoretical specific gravity of the asphalt mixture (dimensionless); $\gamma_{a}$ is the bulk specific gravity of the asphalt mixture (dimensionless). These indicators can be obtained through experimental tests.

A variable *i* was introduced to represent the *i*-th compaction of both sides. According to Equation (1), the compaction degree of the *i*-th compaction ($K_{i}$) can be written as follows:

(2) $$K_{i}=\frac{\gamma_{i}}{\gamma_{s}}\times 100\%$$

where $\gamma_{i}$ is the bulk specific gravity of the asphalt mixture after the *i*-th compaction time (dimensionless).

The change in compaction degree directly reflects the change in void ratio, and there is a relationship between compaction degree (K) and void ratio (VV) as follows:

(3) K+VV=100%

where VV is the measured void ratio of the asphalt mixture (%); there is a negative correlation between K and VV.

#### 2.2.5. Calculation of Void Features in Digital Images

To characterize the void ratio of CT-scanned specimens, the scanning void ratio ($VV_{sm}$) was introduced: it refers to the total area of voids to the total area of cross-section in any cross-section of the specimen, and the relationship is as follows:

(4) $$VV_{sm}=\frac{S_{VV}}{S}\times 100\%$$

(5) $$S_{VV}=\sum_{i=1}^{n}P_{i}$$

where $VV_{sm}$ is the scanning void ratio (%) within any single cross-section of the specimen, $S_{VV}$ is the total area of voids within any scanned cross-section, S is the total area of any scanned cross-section of the specimen (${\mathrm{mm}}^{2}$), $P_{i}$ is the area of any single void in a cross-section (${\mathrm{mm}}^{2}$), n is the number of voids in a single cross-section. These variables can be obtained through software measurement after image processing.

To characterize the average void ratio of the scanned specimen ($\bar{VV_{sm}}$), the average value of the void ratio of all cross-sections in the entire scanned specimen was calculated as follows:

(6) $$\bar{VV_{sm}}=\frac{\sum_{i=1}^{m}VV_{sm}}{m}$$

where m is the number of cross-sections in the scanned specimen.

## 3. Results and Discussion

### 3.1. Analysis of Compaction Evolution

**Figure 5:**
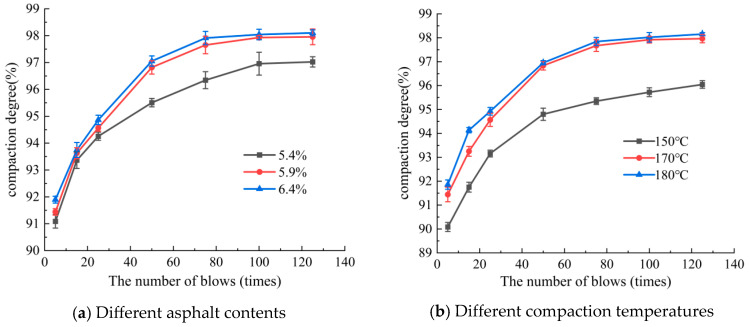
Compaction curve graphs of SMA-13.

To investigate the compaction properties of SMA-13, compaction curves were obtained with varying asphalt content (5.4%, 5.9%, and 6.4%) and initial compaction temperatures (150 °C, 170 °C, and 180 °C). The resulting compaction curves are illustrated in Figure 5.

As shown in Figure 5, the compaction curves of SMA-13 exhibited an exponential growth trend. As the number of blows increased, the compaction degree gradually increased. The following observations can be made:

#### 3.1.1. Impact of Asphalt Content

At a 5.9% asphalt content (optimal content), the compaction curve exhibited the steepest rise, indicating rapid densification and superior compaction efficiency. A lower asphalt content (5.4%) resulted in reduced lubrication between aggregates, leading to slower compaction progression. A higher asphalt content (6.4%) caused overfilling of voids and easily led to oil bleeding, marginally decreasing resistance to further compaction.

#### 3.1.2. Effect of Initial Compaction Temperature

At 170 °C (optimal temperature), SMA-13 achieved the optimum compaction degree due to optimal asphalt viscosity and aggregate mobility. Lower temperatures (150 °C) increased asphalt viscosity, hindering particle rearrangement and reducing compaction effectiveness. Elevated temperatures (180 °C) accelerated asphalt aging, slightly diminishing long-term durability despite comparable compaction performance.

The compaction curve shows an exponential growth trend, and with an increase in the number of blows, the compaction degree gradually increases exponentially. As the asphalt content increased, the more voids filled with asphalt, the denser the asphalt mixture, and the greater the compaction degree. When the optimal asphalt content was exceeded, the increase in the compaction degree was small, and the void overfilled, even leading to oil bleeding. As the initial compaction temperature increased, the porosity decreased. When the initial compaction temperature was higher, the lubrication effect was better, the mixture was compacted easily, and the compaction degree was greater.

This analysis demonstrated that both asphalt content and initial compaction temperature critically influence the compaction evolution of SMA-13, with optimal parameters yielding the most efficient densification process. In summary, the compaction curve of SMA-13 during the compaction process has a mathematical significance, which can reflect the relationship between the number of blows, void ratio, and compaction degree.

### 3.2. Compaction Numerical Regression Model and Compaction Coefficient

#### 3.2.1. Compaction Numerical Regression Model

**Figure 6:**
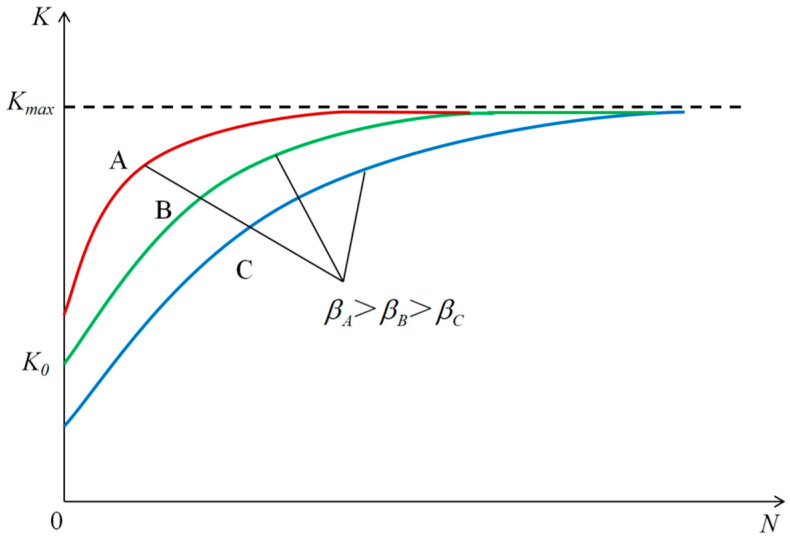
Geometric characteristics and boundary conditions of the compaction curve.

As shown in Figure 5, the compaction curve of SMA-13 under different asphalt contents and compaction temperatures generally increased exponentially. To quantitatively analyze the variation law of compaction, numerical regression analysis was conducted on the compaction curve, and its geometric characteristics and boundary conditions are shown in Figure 6.

In Figure 6, A, B, and C represent the compaction curves of different initial compaction degrees, $K_{0}$ is the initial compaction degree of the mixture (%), $K_{\max}$ is the ultimate compaction degree after the compaction curve converges (%), and $\beta_{A}$, $\beta_{B}$ and $\beta_{C}$ are the curvature factors of different compaction curves, respectively, and N is the number of blows (times).

As shown in Figure 6, the compaction degree of SMA-13 is a univariate exponential function with the number of blows as the variable. Let the exponential regression equation of the curve be:

(7) $$K=\alpha e^{-\beta /N}$$

where α, β is the regression parameters, K is the compaction degree (%), 0<K<100% and N≥0.

When the compaction times is 0, there is a natural accumulation compaction degree (i.e., initial compaction degree) in the asphalt mixture. Therefore, Equation (7) was modified:

(8) $$K=\alpha e^{-\beta /(N+1)}$$

When N→0, thus $K\to K_{0}$ ($K_{0}$ is the initial compaction degree of the mixture), when N→∞, thus $K\to K_{\max}$ ($K_{\max}$ is the maximum compaction degree for the limit), Substituting formula (8) yields:

(9) $$K_{0}=\alpha e^{-\beta }$$

(10) $$K_{\max}=\alpha$$

where the parameter (α) reflects the maximum compaction degree for the limit, and the curvature factor (β) reflects the increased magnitude of compaction degree.

Using Equation (8), a numerical regression analysis was performed on the compaction curves of SMA-13. To distinguish between different types, the SMA-13 specimens with an asphalt content of 5.4%, 5.9%, and 6.4% were labeled as SMA-13(5.4), SMA-13(5.9), and SMA-13(6.4), respectively. Similarly, the specimens with initial compaction temperatures of 150 °C, 170 °C, and 180 °C were labeled as SMA-13(150), SMA-13(170), and SMA-13(180), respectively. The regression parameters for these compaction curves are summarized in Table 4.

The following observations can be made from Table 4:

SMA-13(5.9) exhibited the highest curvature factor (β = 0.086) and ultimate compaction degree ($K_{\max}$ = 98.22%), which indicated that SMA-13(5.9) had the optimal compaction efficiency. Lower asphalt content (SMA-13(5.4)) resulted in reduced compaction performance, while higher asphalt content (SMA-13(6.4)) showed marginal improvements in compaction performance.

SMA-13(170) achieved the highest compaction degree ($K_{\max}$ = 98.22%) and curvature factor (β = 0.086), which confirmed that 170 °C is the optimal compaction temperature. Lower temperatures (SMA-13(150)) hindered compaction, while higher temperatures (SMA-13(180)) led to smaller gains and potential asphalt aging.

The high correlation coefficient (*R*^2^ > 0.99) proved that this model is correct in describing the compaction behavior of SMA-13. It showed that the results of regression analysis were consistent with the experimental results, and the model is reasonable and effective.

#### 3.2.2. The Evaluation Index: Compaction Coefficient

The compaction characteristics of an asphalt mixture refer to the difficulty of achieving a fixed compaction degree under the same compaction work. As shown in Figure 5 and Figure 6, the compaction characteristics are related to the initial compaction degree $K_{0}$, the ultimate compaction degree $K_{\max}$, and the curvature factor β. In order to evaluate the compaction characteristics of SMA-13 quantitatively, the compaction coefficient C was introduced as an evaluation index, and the formula is as follows:

(11) C=βΔK

(12) $$\Delta K=K_{\max}-K_{0}$$

where ΔK is the difference between the ultimate compaction degree and the initial compaction degree, taken as a numerical value, dimensionless.

The larger ΔK and β, the greater the compaction coefficient C, the easier the compaction of SMA-13, and the better the compaction effect. When the value of C is smaller, it indicates that compaction is more difficult, and the compaction effect is worse. The results of compaction parameters for SMA-13 according to Equation (11) are shown in Table 5.

As shown in Table 5, under the optimal asphalt content (5.9%) and initial compaction temperature (170 °C), the compaction degree difference (ΔK) was the largest, the curvature factor (β) was the highest, and the compaction coefficient (C) reached its maximum value. This indicated the best compaction performance and the easiest compaction. The ranking of compaction difficulty is as follows: SMA-13 (170) < SMA-13 (180) < SMA-13 (150), and SMA-13 (5.9) < SMA-13 (6.4) < SMA-13 (5.4).

The compaction coefficient (C) is closely related to the initial compaction degree ($K_{0}$), ultimate compaction degree ($K_{\max}$), and curvature factor (β). Both asphalt content and initial compaction temperature significantly influence the compaction coefficient. The compaction coefficient (C) effectively characterizes the ease of compaction and the compaction performance of SMA-13. It provides a quantitative measure for evaluating the influence of material composition and process parameters on compaction behavior.

### 3.3. Evolution of Void Characteristics During Compaction

**Figure 7:**
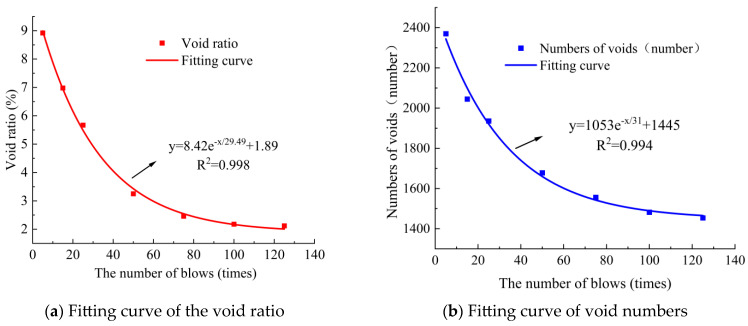
Variation curve of void ratio and void quantity with SMA-13.

**Figure 8:**
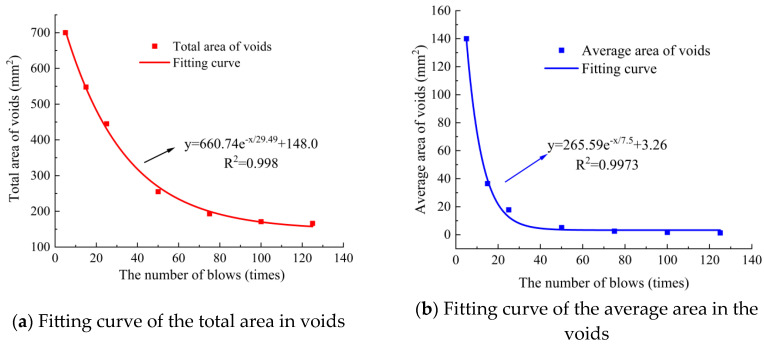
Variation curve of void area with SMA-13.

Using digital image processing techniques, the void characteristics of these specimens with SMA-13 under optimal compaction conditions (initial compaction temperature: 170 °C, asphalt content: 5.9%) were analyzed for different numbers of blows (5, 15, 25, 50, 75, 100, and 125 per face). The void characteristics included void ratio, number of voids, total area of voids, average area of voids, and equivalent diameter of voids. The variation curve of void characteristics during the compaction process was obtained, and nonlinear fitting was performed, as shown in Figure 7 and Figure 8.

As shown in Figure 7, the variation pattern of the number of voids and void ratio during compaction of SMA-13 was similar, the attenuation was faster in the early stage and slower in the later stage. With an increase in the number of blows, the number of voids and void ratio decreased with an exponential decay trend. There was a good correlation among the number of voids, void ratio, and the number of blows, which can be established by fitting an exponential function.

As shown in Figure 8, with an increase in the number of blows, the total area of voids and the average area of voids decreased exponentially. When the number of blows was between 0 and 25, the attenuation rate of voids was the highest, and compaction was the easiest. When the number of blows was between 25 and 75, the average area of the voids decayed slowly, and the compaction difficulty gradually increased. When the number of blows was 75–125, the average area of voids tended to stabilize, and the compaction degree tended to stabilize, making it difficult to compact.

**Figure 9:**
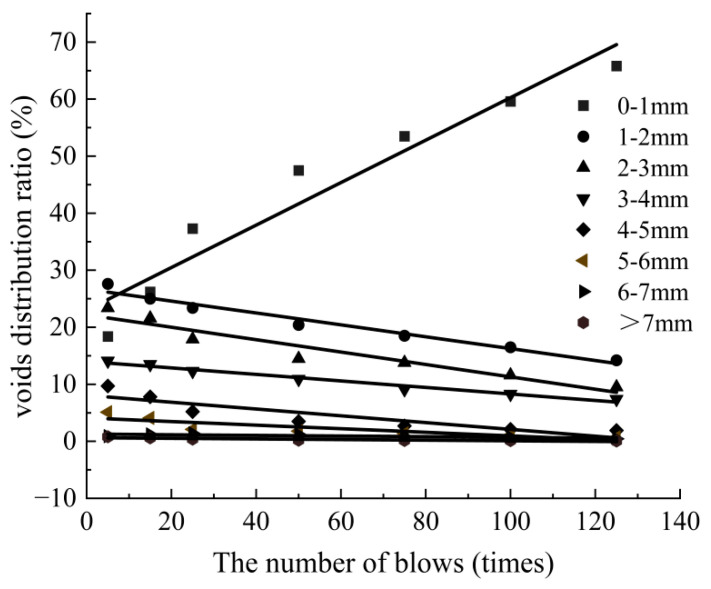
The evolution of the void distribution rate in SMA-13.

To analyze the distribution of voids during compaction of SMA-13, the equivalent diameter of voids was divided into 8 levels: 0–1 mm, 1–2 mm, 2–3 mm, 3–4 mm, 4–5 mm, 5–6 mm, 6–7 mm, and >7 mm. The number of voids in different levels for CT images under different numbers of blows (5, 15, 25, 50, 75, 100, and 125) in SMA-13 was calculated. The void distribution ratio is the number of voids within a certain equivalent diameter range as a percentage of the total number of voids. The evolution of void distribution rate was obtained for different equivalent diameters during compaction, and a linear fit was performed. The results are shown in Figure 9.

As shown in Figure 9, with an increase in the number of blows, the voids with equivalent diameters of 1–7 mm gradually decreased, and the void distribution rate showed a nonproportional linear decay. When the equivalent diameter of voids was greater than 6 mm, the linear decay of the void distribution rate was not significant.

The voids with the equivalent diameter of 0–1 mm gradually increased, and the void distribution rate showed a higher linear growth. The growth rate of voids with the equivalent diameters of 0–1 mm was much higher than the decrease rate of voids with the equivalent diameters of 1–7 mm. The voids with the equivalent diameter of 0–1 mm were the main component of SMA-13. The number of blows was linearly correlated with the distribution rate of voids with different equivalent diameters.

### 3.4. Distribution and Evolution of Void Ratio

The void ratio of an asphalt mixture is a key control indicator for compaction quality, which can effectively evaluate the compaction quality of asphalt mixture and has a significant impact on compaction characteristics.

**Figure 10:**
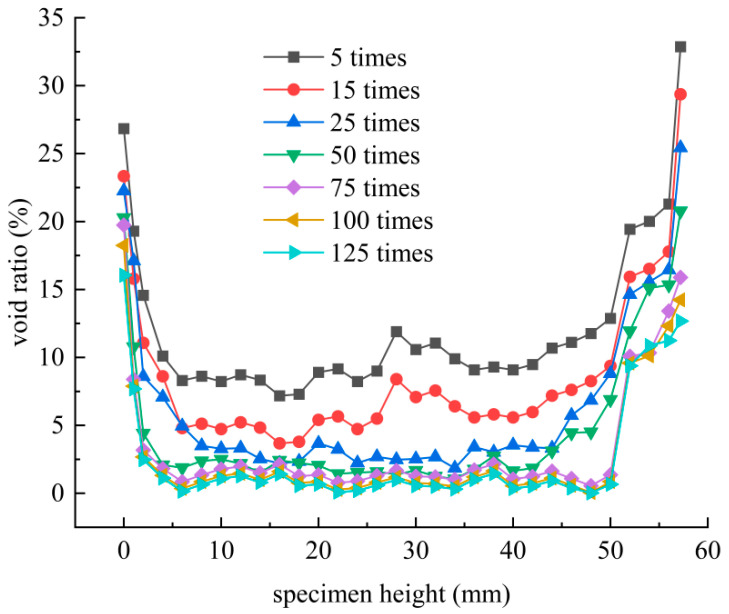
Void ratio of SMA-13 along the height direction.

Therefore, the void ratio along the height direction of the specimen was analyzed based on CT images. Statistical analysis was conducted along the height direction of the specimen to obtain the void ratio of SMA-13 at different number of blows (5, 15, 25, 50, 75,100, and 125), and the height of the specimen started counting from the bottom surface as 0, as shown in Figure 10.

As shown in Figure 10, under different compaction times, the void ratio of SMA-13 exhibited a distribution characteristic of “great at both ends and small in the middle”. With an increase in the number of blows, the void ratio gradually decreased, especially at the height of 5–50 mm in the specimen, which decreased from about 10% to about 0% and was distributed evenly. The void ratio at the bottom 0–5 mm and top >50 mm was large and distributed unevenly, mostly between 10% and 40%.

During the compaction process, the void ratio of the bottom and top was much higher than that of the middle. It is shown that the void ratio at the bottom and top during compaction is strongly influenced by external factors such as bottom constraints, compaction machinery, climate, self-weight, and leveling during compaction.

## 4. Conclusions

- The compaction curve of SMA-13 increases exponentially during compaction, and the established numerical regression model of compaction can better characterize the compaction characteristics of SMA-13. The compaction characteristics of SMA-13 are significantly correlated with the initial compaction degree ($K_{0}$), ultimate compaction degree ($K_{\max}$) and curvature factor (β). The compaction coefficient (C) can effectively evaluate the compaction difficulty of SMA-13. When the compaction coefficient is larger, SMA-13 is easier to compact, and the compaction effect is better.
- The void ratio, number of voids, and void area of SMA-13 during compaction show exponential function decay and exponential function correlation with the number of blows of compaction. At the early compaction (0–25 times), the compaction degree grows fastest, and it is easiest to compact, but it becomes more and more difficult to compact in the middle and late stages. Therefore, the construction should pay special attention to the initial compaction, which can obtain better compaction performance.
- During compaction, the voids with equivalent diameters of 1–7 mm gradually decreased in SMA-13, and the distribution rate of the voids showed a non-equal proportional linear decay; the voids with equivalent diameters of 0–1 mm gradually increased, and the distribution rate increased linearly, and they were the main void components of SMA-13.
- During compaction, the void ratio of SMA-13 gradually decreased along the direction of height, and the distribution of void ratio was “great at both ends and small in the middle”. The void ratio at 5–55 mm decreased from about 10% to about 0%, and the void ratio distribution was relatively uniform. The void ratio at the bottom 0–5 mm and top >50 mm was large and unevenly distributed, mostly between 10% and 40%.
- It is worth noting that the compaction coefficient (C) in the paper only evaluates the compaction difficulty of SMA-13, and more asphalt mixture types (e.g., AC-13, AC-20, etc.) should be covered in later studies to explore the reliability and adaptability of the compaction coefficient (C) to other mixtures.

## Figures and Tables

**Table 1:** Technical properties of SBS modified asphalt.

Property		Test Result	Technical Requirement
Penetration (25 °C, 5 s, 100 g, 0.1 mm)		57	40–60
Softening point T_R&B_ (°C)		65.6	≥60
Ductility (5 °C, 5 cm/min)		26.8	≤20
Brinell rotational viscosity (135 °C, Pa s)		1.38	≤3
Density (25 °C, g/cm^3^)		1.023	-
Specific Gravity (Relative to water density at 25 °C, dimensionless)		1.026	-
After RTFOT	Mass change (%)	0.02	≤±1.0
	Needle penetration ratio (25 °C,%)	68.43	≥65
	Ductility (5 °C, cm)	17.3	≥15

**Table 2:** Technical properties of aggregates and mineral powder.

Aggregate Grade (Basalt Gravel)	Apparent Specific Gravity (Dimensionless)	Apparent Density (g/cm^3^)	Bulk Specific Gravity (Dimensionless)	Water Absorption (%)
9.5–16 mm	2.938	2.931	2.883	0.65
4.75–9.5 mm	2.937	2.930	2.858	0.94
2.36–4.75 mm	2.803	2.796	2.703	1.32
0–3 mm	2.712	2.704	-	-
Mineral powder	2.75	2.745	-	-

**Table 3:** Aggregate gradation.

Asphalt Mixture Type	Mass Percentage (%) Passing the Following Sieve Sizes (mm)									
	0.075	0.15	0.3	0.6	1.18	2.36	4.75	9.5	13.2	16
SMA-13	10	12.9	14.7	17	19	21.4	27.6	60.8	96.8	100
Upper limit	12	15	16	20	24	26	34	75	100	100
Lower limit	8	9	10	12	14	15	20	50	95	100

**Table 4:** Compaction parameter values of SMA-13.

SMA-13 Type	Initial Compaction Degree (%) $K_{0}$	Ultimate Compaction Degree (%) $K_{max}$	Curvature Factor β	Correlation Coefficient $R^{2}$
SMA-13(5.4)	89.86	96.46	0.071	0.9905
SMA-13(5.9)	90.12	98.23	0.086	0.9982
SMA-13(6.4)	90.62	98.32	0.082	0.9967
SMA-13(150)	88.93	96	0.076	0.9978
SMA-13(170)	90.11	98.22	0.086	0.9982
SMA-13(180)	91.92	98.5	0.069	0.9947

**Table 5:** Calculation results of compaction parameters for SMA-13.

SMA-13 Types	Compaction Degree Difference ΔK	Curvature Factor β	Compaction Coefficient C
SMA-13(5.4)	6.60	0.071	0.47
SMA-13(5.9)	8.11	0.086	0.70
SMA-13(6.4)	7.7	0.082	0.63
SMA-13(150)	7.07	0.076	0.54
SMA-13(170)	8.11	0.086	0.70
SMA-13(180)	7.56	0.080	0.61

## Data Availability

All data generated or analysed during this study are included in this published article.
